# Revisions of One Anastomosis Gastric Bypass with Biliopancreatic Limb of 250 cm Multicentre Retrospective Study with a Mean Follow-up of 11 years

**DOI:** 10.1007/s11695-026-08869-x

**Published:** 2026-07-25

**Authors:** ABDULZAHRA Hussain, Alaa AlWadees, Samer AL Hakkak, Tamer Habeeb, Peter Vasas

**Affiliations:** 1 University of Alkafeel, Najaf, Iraq; 2 Homerton University Hospital, London, United Kingdom; 3 Jabir Ibn Hayyan University for medical and pharmaceutical sciences, Najaf, Iraq; 4 Zagazig University, Zagazig, Egypt; 5 Doncaster and Bassetlaw Teaching Hospitals NHS Foundation Trust, Doncaster, United Kingdom; 6 College of medicine, Semmelweis University, Budapest, Hungary

## Abstract

**Background:**

One-anastomosis gastric bypass (OAGB) is a widely performed bariatric procedure. The length of the biliopancreatic limb (BPL) is a key determinant of weight loss and metabolic effect, but excessively long limbs may predispose to malabsorptive complications. Long-term outcome data for fixed long BPLs remain limited.

**Objective:**

To evaluate long-term severe complications requiring revisional surgery following OAGB with a fixed biliopancreatic limb length of 250 cm.

**Methods:**

This multicentre retrospective cohort study included all consecutive patients who underwent primary OAGB with a 250-cm BPL between 2010 and 2020. Data were extracted from hospital electronic and paper records. Patients were invited for a structured clinical and biochemical assessment. Severe complications were predefined and reviewed by a multidisciplinary team. Outcomes of interest were severe complications requiring revisional surgery. Follow-up ranged from 7 to 15 years.

**Results:**

Of 892 OAGB procedures performed during the study period, 97 patients (10.9%) underwent OAGB with a 250-cm BPL. Complete follow-up data were available for 78 patients (80.4%). Mean age was 47 years, mean preoperative BMI was 46.6 kg/m^2^, and 75.3% were female. Nineteen patients (24.3%) developed severe complications requiring revisional surgery. Indications included severe protein malnutrition (5.1%), refractory bile or acid reflux (6.4%), severe diarrhoea (6.4%), liver failure (2.5%), twisted gastrojejunostomy (2.5%), and excessive weight loss (1.3%). Revisions included conversion to sleeve gastrectomy, Roux-en-Y gastric bypass, or reversal to normal anatomy.

**Conclusion:**

OAGB with a fixed biliopancreatic limb length of 250 cm was associated with a high rate of severe long-term complications requiring revisional surgery. These findings support cautious tailoring of BPL length and avoidance of routinely long limbs in OAGB.

## Key points

A fixed biliopancreatic limb length of 250 cm was associated with a high incidence of severe late complications requiring revisional surgery.

The main complications were malabsorptive, including protein–calorie malnutrition, chronic diarrhoea, liver failure, and refractory gastro-oesophageal reflux.

Standardised long-term follow-up protocols are required to identify and manage severe complications of OAGB with 250cm BPL.

## Introduction

One-anastomosis gastric bypass (OAGB), previously known as mini-gastric bypass (MGB), was first described by Rutledge in 1997 and has since evolved into a widely performed bariatric procedure worldwide [1]. Early reports demonstrated acceptable safety profiles and effective weight loss, contributing to its increasing adoption. According to the most recent International Federation for the Surgery of Obesity and Metabolic Disorders (IFSO) registry, OAGB is currently the third most commonly performed bariatric and metabolic operation in certain regions [2].

The surgical technique of OAGB involves creating a long, narrow gastric pouch and a single gastrojejunostomy to a biliopancreatic limb (BPL) of variable length. Compared with Roux-en-Y gastric bypass (RYGB), OAGB avoids a jejunojejunostomy and internal defect closures; is technically less complex and is associated with shorter operative time and learning curve [3–9]. These features have supported its use both as a primary and revisional bariatric procedure [8–10].

The metabolic and weight-loss effects of OAGB are largely driven by the length of the biliopancreatic limb, which determines the degree of intestinal exclusion from nutrient absorption [10–12]. While longer BPLs may enhance weight loss and metabolic outcomes, they are also associated with an increased risk of protein–calorie malnutrition, micronutrient deficiencies, chronic diarrhoea, steatorrhoea, and liver dysfunction [13–17]. Similar complications have been reported following other malabsorptive bariatric procedures when the excluded limb is excessive, or the common channel is critically short [18–20].

Concerns have also been raised regarding bile reflux after OAGB and its potential impact on the gastric pouch and lower oesophageal mucosa [21]. Although OAGB differs fundamentally from historical loop gastrojejunostomy techniques,like Mason’s one, bile reflux remains a recognised, long-term postoperative issue, particularly in susceptible patients. Consequently, careful patient selection is essential, and OAGB is generally avoided in individuals with significant preoperative gastro-oesophageal reflux disease, significant hiatus hernia, Barrett’s oesophagus, or a strong family history of upper gastrointestinal malignancy [22–26].

Randomised controlled trials and large observational studies have demonstrated that OAGB is not inferior to RYGB in terms of weight loss and metabolic improvement [27, 28]. However, these studies have also reported higher incidences of diarrhoea, nutritional adverse events, and bile reflux, particularly when longer (> 250 cm) biliopancreatic limbs were used [27–30]. More recent consensus statements and expert recommendations emphasise the importance of tailoring BPL length to patient characteristics and avoiding excessive malabsorption [22, 24].

Despite this, there remains no universally accepted standard for optimal biliopancreatic limb length, and long-term outcome data for long BPLs are limited. In particular, the incidence and nature of late severe complications requiring revisional surgery after OAGB with a biliopancreatic limb length of 250 cm are poorly defined and not well reported.

The aim of this multicentre retrospective study was therefore to evaluate the long-term severe complications associated with OAGB performed with a fixed biliopancreatic limb length of 250 cm, with a specific focus on complications necessitating revisional surgery over extended follow-up.

## Methodology

### Study Design and Setting

A multicentre, retrospective cohort study was designed and completed, evaluating the long-term severe complications following one-anastomosis gastric bypass (OAGB), performed with a long biliopancreatic limb (BPL) length of 250 cm. The included data are provided by 5 centres and 5 surgeons. The study included patients who had the OAGB (primary procedure) between January 2010 and December 2020, during a period when routine measurement of total small bowel length and common channel was not part of institutional practice. However, this study involved the revisions (secondary procedure) performed between XX and YY. The participating centres were tertiary bariatric surgery units performing high volumes of bariatric and metabolic surgery and all procedures were completed by fellowship-trained consultant surgeons.

### Patient Selection

All consecutive adult patients who underwent primary OAGB with a biliopancreatic limb length of 250 cm during the study period were eligible for inclusion. Patients who underwent OAGB with shorter or tailored BPL lengths were excluded. Revisional OAGB procedures were also excluded.

The original criteria for selecting 250 cm for BPL were:

- Patients who were older than 18 years old and less than 69 year.
- The body mass index was ≥ 40 kg/m^2^.
- The patient agrees to have such strong metabolic surgery and is committed to a long and intensive follow-up regimen.

Eligible patients were identified from institutional operative databases. Demographic data, operative details, and follow-up information were obtained from electronic patient records and archived case notes.

### Baseline Assessment

Baseline characteristics included age, sex, preoperative body mass index (BMI), and year of surgery. BMI was calculated as weight in kilograms divided by height in metres squared (kg/m^2^). Preoperative assessment followed standard bariatric pathways and included multidisciplinary evaluation in accordance with national guidelines in place at the time of surgery.

#### Operative technique

General anaesthesia, reverse Trendelenburg, French position. Pneumoperitoneum was created by the Veress needle, and the Nathanson liver retractor was routinely used. Standard technique of a 12–15 cm gastric pouch was created over a 34–36 FR bougie and an end-to-side linear stapler anastomosis was created with small bowel at a distance of 250 cm from the duodeno-jejunal junction DJJ.The enterotomy was closed in two layers with absorbable sutures. A routine leak test was performed, and no drains were left in the operative field unless concerns of bleeding, difficult procedure or previous laparotomy and adhesions.

#### Pre-revision optimisation strategies

Perioperative nutritional optimisation strategies were systematically implemented before revisional surgery. Enteral and parenteral nutrition protocols were implemented. 7 patients had a gastrostomy feeding tube inserted in the remnant stomach. No revisional surgery was performed unless the albumin level was > 25 g/dL.

### Follow-up and Clinical Assessment

All eligible patients were invited to attend dedicated follow-up clinics for structured assessment. During each visit, patients underwent a comprehensive clinical evaluation, including history, physical examination, and targeted investigations guided by presenting symptoms. Routine laboratory investigations included full blood count, renal function, liver function tests, serum albumin, total protein, iron studies, vitamin levels, trace elements, and bone profile. Additional investigations were performed as clinically indicated:

- Patients presenting with chronic diarrhoea underwent exocrine pancreatic enzyme testing, colonoscopy with colonic and ileal biopsies to exclude alternative pathology.
- Patients presenting with reflux symptoms underwent upper gastrointestinal endoscopy, barium swallow studies, Helicobacter pylori testing, 24 h oesophageal pH monitoring, and manometry where appropriate.

### Definition of Complications and Outcomes

Complications were categorised using a predefined institutional proforma into “mild”, “moderate”, or “severe” based on clinical impact, biochemical derangement, and need for intervention. The primary outcome of the study was the occurrence of severe complications requiring revisional surgery.

- Protein–calorie malnutrition refractory to medical- and nutritional management: **defined as a total serum protein value lower than 63 g/l, and a total albumin value lower than 35 g/l with presence or absence of peripheral oedema, ascites or pleural effusion, depending on severity.**
- Severe or decompensated liver dysfunction is **defined as abnormal protein, bilirubin and liver enzyme levels**
- Refractory bile- or acid reflux significantly impairing quality of life: **defined as daily reflux symptoms that are refractory to medications and a change of lifestyle. Objective tests of gastroscopy diagnose the reflux [must bile present in the gastric pouch and or oesophagus, with a grade of oesophagitis] plus confirmed reflux on barium study. These two tests are further supported by a pH and manometry study to confirm reflux and assess the manometry characteristics in each patient.**
- Chronic debilitating diarrhoea is **defined as bowel motion that has significantly changed after the operation, and it is more than 3 times of loose stool per day.**
- Excessive weight loss is **defined as more that 100% loss of excess weight.**
- Mechanical complications such as twisted or obstructed gastrojejunostomy are **defined as obstruction symptoms of vomiting, abdominal pain, distension, and constipation.**

### Management and Revision Strategy

All patients diagnosed with severe complications were discussed in a dedicated bariatric multidisciplinary team (MDT) meeting. Management decisions were individualised and included continued nutritional and medical therapy, revisional bariatric surgery, or reversal to normal anatomy, depending on the nature and severity of the complication. Revisional procedures included conversion to Roux-en-Y gastric bypass, sleeve gastrectomy, or restoration of normal gastrointestinal anatomy. The choice of procedure was guided by the presenting pathology and MDT consensus.

Our management pathway was as follows:

Patients with liver failure and excessive weight loss were revised to normal anatomy due to the life-threatening nature of their condition. We consider this approach universally recommended in such critical scenarios.

Severe protein malnutrition was closely associated with BPL length [colonoscopy and biopsies revealed no mucosal abnormalities] or the length of the common channel where absorption occurs. These patients were revised to LSG to maintain a restrictive effect while preventing significant long-term weight regain. In mild -moderate hypoalbuminemia, long-term follow-up is essential to monitor protein levels, as these patients remain at risk of severe hypoproteinaemia during acute illness, bowel resections, or gastrointestinal inflammatory or ischemic events. Nutritional management, including a high-protein diet, dietary follow-up, and adherence to British Obesity and Metabolic Surgery Society (BOMSS) guidance, is critical. Four patients with severe protein–caloric malnutrition were revised to LSG, whereas 23 of 78 patients (29%) developed mild-to-moderate protein malnutrition and did not require revisions.

### Follow-up Completeness and Loss to Follow-up

Extensive efforts were made to maximise follow-up attendance. Patients who did not attend scheduled appointments were contacted by telephone and letter, and contact details were cross-checked against central hospital records. Patients who could not be contacted after repeated attempts were classified as lost to follow-up. We tried to include all patients who developed complications and needed revisions in this study. This by no means exclusively includes all the patients. Nearly 20% loss to follow up might indicate that some patients might be treated at another centres or did not develop complications, which may lower the incidence rate of revisions.The follow up period was 7–15 years (with a mean of 11 years) from the primary operations.

### Statistical Analysis

Data were analysed descriptively. Continuous variables are presented as means with standard deviations or medians with ranges, as appropriate. Categorical variables are presented as frequencies and percentages.Data analysis was conducted using standard statistical software (IBM SPSS).

### Ethical Considerations

The study was conducted in accordance with institutional governance policies for retrospective clinical evaluation and audit. All data were anonymised before analysis.

## Results

### Study Population

Between January 2010 and December 2020, a total of **892 one-anastomosis gastric bypass (OAGB)** procedures were performed across the participating centres. Of these, **97 patients (10.9%)** underwent primary OAGB with a fixed biliopancreatic limb (BPL) length of **250 cm** and were eligible for inclusion.

Complete long-term follow-up data were available for **78 patients (80.4%),** who constituted the final study cohort. Nineteen patients (19.6%) were lost to follow-up despite repeated contact attempts.

The mean age of the analysed cohort was **47 ± 10.5 years**, and **73 patients (75.3%)** were female. The mean preoperative body mass index (BMI) was **46.6 ± 7.1 kg/m**^**2**^**,** with a range of **31.0–73.2 kg/m**^**2**^**.** Baseline demographic and clinical characteristics are summarised in Table 1**.**

**Table 1:** Baseline patients’ characteristics

Characteristic	Value
Age, mean ± SD (years)	47 ± 10.5
Female sex, n (%)	73 (75.3)
Preoperative BMI, mean ± SD (kg/m^2^)	46.6 ± 7.1
BMI range (kg/m^2^)	31.0–73.2
BPL length	250 cm
Follow-up duration, median (range)	11 years (7–15)

Severe Complications Requiring Revisional Surgery:

The definition of the severity of complications is highlighted in Table 2.

**Table 2:** Definition of the Severity of post-bariatric surgery outcomes

Complication	Mild	Moderate	Severe
**Malnutrition** **(albumin level)**	30-37gms/100 ml	25–29 gms/100 ml	< 25 gms/100 mls
**Malnutrition (patient-reported factors)**	Often asymptomatic or subtle signs (fatigue, mild hair loss) May have mild muscle wasting	More obvious muscle wasting (especially extremities) Fatigue, weakness, sometimes mild oedema	Peripheral oedema; muscle wasting or generalized body weakness;Hair loss > 2 teeth crumbled or fallen out
**Reflux**	• Occasional heartburn or regurgitation • Symptoms are infrequent (e.g., less than once per week)	Heartburn or regurgitation occurs **more frequently** (e.g., weekly or several times a week) Symptoms **may interfere with daily activities** May require **regular acid-suppressive therapy**	vomiting uncontrolled with proton pump inhibitor (PPI) therapy Limited diet; able to tolerate only a few foods
**Weight loss** %EWL: within 6–12 months post-surgery	25–50%	50–70%	Body mass index (BMI) < 18.5 kg/m^2^ post-operatively > 20% excess body weight loss within the first 8 weeks post-operatively > 100% excess body weight loss at 18 months;
**Diarrhoea – frequency**	1–2 episodes per day	2–5 episodes per day	≥ 5 episodes per day Life-limiting symptoms Change of work or social life, stress

During follow-up, **19 patients (24.3%)** developed **severe complications requiring revisional surgery.** The indications for revision and corresponding management strategies are detailed in Table 3.

**Table 3:** Complications of OAGB and management

Complication	n (%)	Revision strategy
Severe reflux	5 (6.4)	Conversion to RYGB
Severe diarrhoea	5 (6.4)	Conversion to LSG
Severe protein malnutrition	4 (5.1)	Conversion to LSG
Liver failure	2 (2.5)	Reversal to normal anatomy
Twisted gastrojejunostomy	2 (2.5)	Surgical correction
Excessive weight loss	1 (1.3)	Reversal to normal anatomy
**Total revisions**	**19 (24.3)**	—

**Fig. 1:**
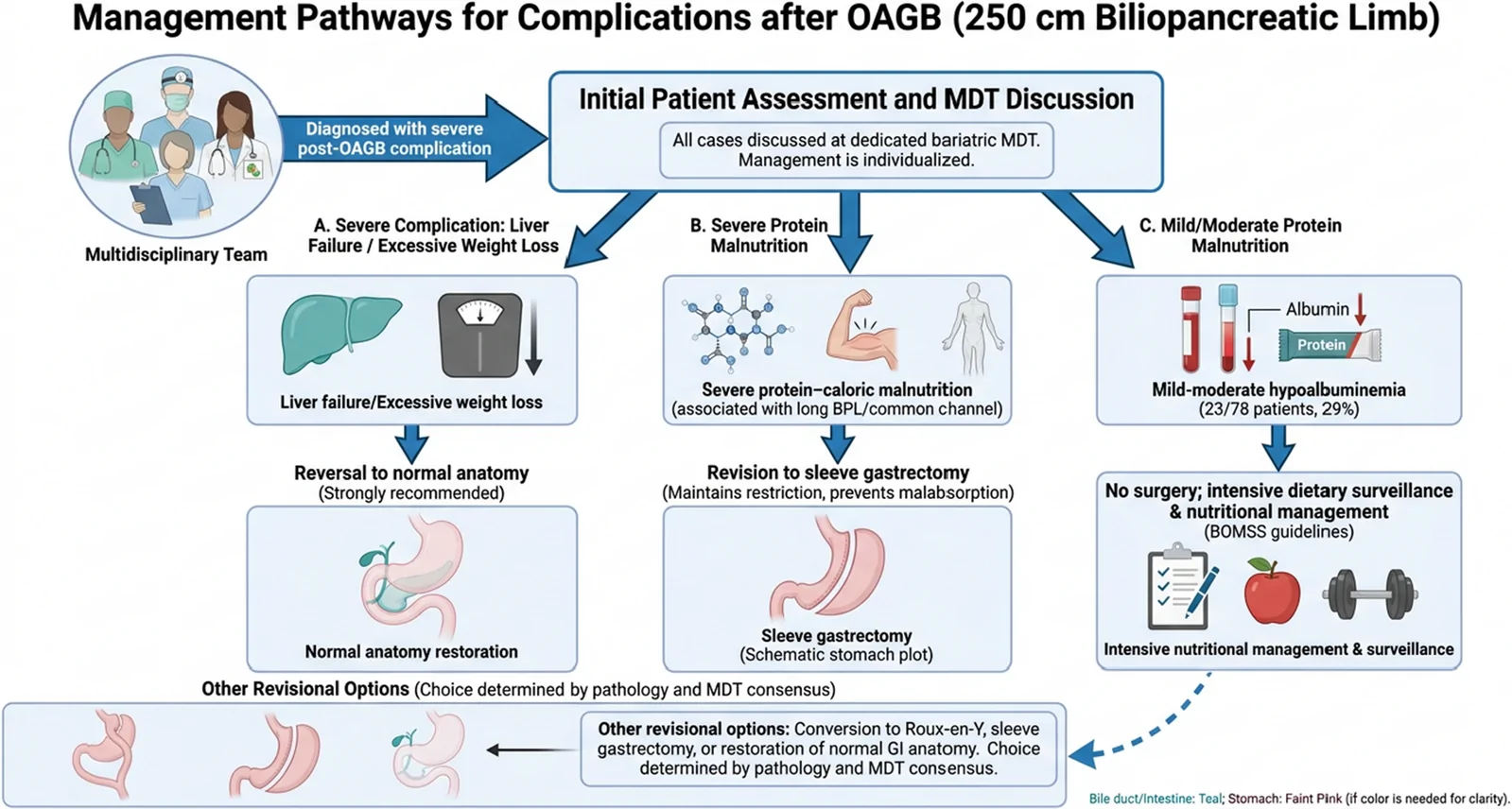
Time to revisions

**Fig. 2:**
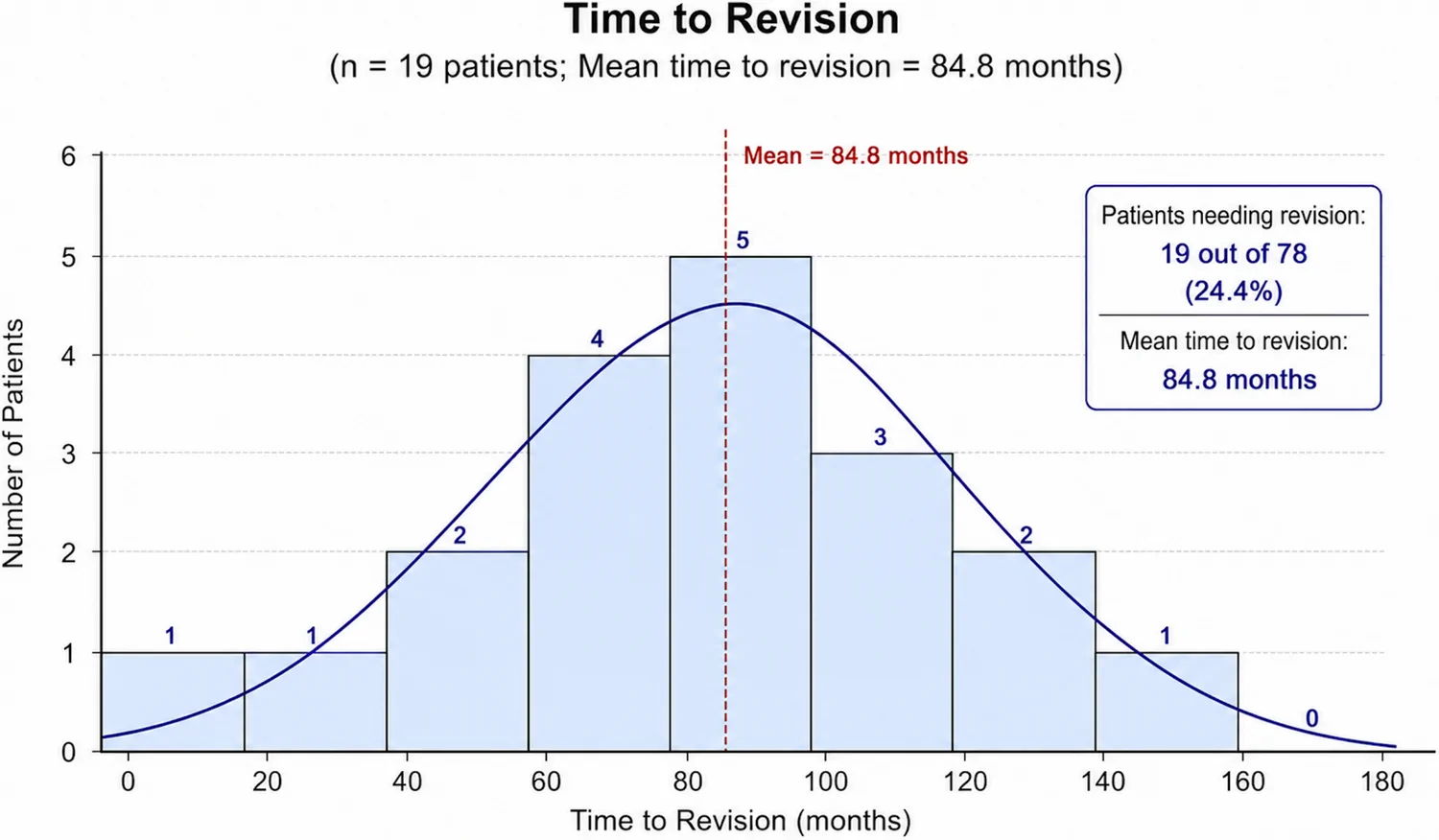
Management pathways

All the decisions of revisions were taken by the bariatric MDT, depending on the clinical data and the the decisions were discussed with the patients for final decision of management. Figure 1 shows the times to revisions and Fig. 2 shows the management pathways.

### The most frequent indications for revision were:

- Severe refractory reflux in 5 patients (6.4%)**,** characterised by persistent symptoms despite maximal medical therapy and significant impairment of quality of life. All patients underwent conversion to Roux-en-Y gastric bypass**.**
- Severe chronic diarrhoea in 5 patients (6.4%)**,** unresponsive to dietary and medical management, with exclusion of alternative gastrointestinal pathology. These patients underwent revision to sleeve gastrectomy**.**
- Severe protein–calorie malnutrition in 4 patients (5.1%)**,** presenting with recurrent hospital admissions, oedema, ascites, pleural or pericardial effusions, and profound hypoalbuminaemia. These patients were revised to sleeve gastrectomy following multidisciplinary review and pre-operative nutritional optimisation
- Liver failure in 2 patients (2.5%)**,** both presenting with decompensated cirrhosis (Child–Pugh class C) and marked derangement of liver function tests. These patients underwent reversal to normal anatomy**.**
- Mechanical complications**,** including twisted gastrojejunostomy, occurred in 2 patients (2.5%) and were managed surgically. The twisted gastrojejunostomy was most likely because of a technically suboptimal primary procedure.
- Excessive weight loss occurred in 1 patient (1.3%), who underwent reversal to normal anatomy**.**

#### Nutritional Outcomes

In addition to patients requiring revision, **23 patients (29.5%)** developed **mild to moderate protein deficiency** during follow-up, managed with dietary optimisation and nutritional supplementation. These patients did not require revisional surgery but required prolonged dietary surveillance.

#### Follow up

All patients included in the final analysis underwent structured clinical and biochemical assessment in dedicated follow-up clinics. A total of 78 (80.4%) patients were examined and assessed in the clinic. Patients who developed severe complications were assessed by clinical and laboratory investigations and referred to the multidisciplinary team MDT for review before a decision was made. Total follow-up is 7–15 years with a median follow-up of 11 years. Lost to follow-up 19(19.6%) patients. The total available data for analysis were for 78 (80.4%) patients.

## Discussion

The most significant finding of our study was the high incidence of the need for revisional surgery in patients with a biliopancreatic limb (BPL) of 250 cm, reaching 24.3%. Many previous OAGB studies have not reported this incidence, likely due to reporting bias or mixed case series that diluted the true number of revisions in long BPL cases as well as the short term follow up. In our cohort, 19 (24.3%) severe complications were identified, and all management decisions were made by a multi-disciplinary team.

Reflux-related complications and revisions were higher than those reported in other studies, likely due to patient-specific factors such as hiatus hernia, dietary habits, social milieu, or technical aspects of the procedure, rather than BPL length alone [31, 32]. OAGB can induce de novo reflux or worsen pre-existing bile reflux. We think patients with preoperative > 2 cm hiatus hernia, high-dose proton pump inhibitor use, Barrett’s oesophagus, or family history of upper GI cancer risk should generally not undergo OAGB. While simultaneous hiatus hernia repair and OAGB may be an option in selected patients, randomised trial data are lacking, and existing evidence is limited to self-reported, level 4 retrospective studies.

Currently, there are no national or international guidelines for long-term management of OAGB complications. Most available publications are retrospective and prone to reporting bias. Notably, Lee et al. conducted a systematic review of > 7,000 OAGB procedures, reported 3.4% major complications needed significant management and revisions and 0.14% mortality [23].

Technical factors such as inadequate pouch creation (< 12 cm), anastomotic rotation, inappropriate BPL length, GJ stoma size discrepancies, and poor patient selections significantly influence outcomes of the OAGB [1, 24–26].

The critical question remains: what BPL length achieves optimal weight loss without compromising long-term physiology? Randomised controlled trials comparing OAGB to other procedures, including the French YOMEGA trial, concluded that OAGB is not inferior to RYGB regarding weight loss and metabolic improvement at 2 years. However, longer BPLs (200 cm) were associated with higher rates of diarrhoea, steatorrhea, and nutritional adverse events [27].

In the YOMEGA trial, adverse malnutrition events occurred in nine (7.8%) OAGB patients, with four (3.4%) undergoing revision surgery, while none were revised in the first Taiwanese OAGB RCT [28]. Bile reflux was observed, but did not affect the quality of life or revision rates. Similarly, the UK’s largest OAGB study (925 patients) reported 2.3% major complications, zero 30-day mortality, and 0.2% loss to follow-up over 2.5 years, suggesting an optimal BPL of < 200 cm [30]. The UK MGB collaborative study included a heterogeneous group of patients with different BPL.

Carbajo et al. (Spain) reported 1,200 patients with BPL 250–350 cm [33] and a 0% revision rate, using a midpoint calculation based on total small bowel length. Despite using a comparable BPL, our revision rates were substantially higher at 24%. Long-term follow-up studies with BPL 200–250 cm demonstrated lower revision rates: major malnutrition requiring conversion to normal anatomy (1.3%) and revision to RYGB for reflux (5.7%) [34]. Carbajo’s 6–12 year follow-up reported only minor complications, none requiring revision [35]. Ten-year follow-up of 156 patients with 200–250 cm BPL showed a 0.7% revision rate for reflux and severe malnutrition [36]. The YOMEGA 5-year outcomes showed 8% conversions to RYGB, including 40% reporting reflux [37]. A cohort of 405 OAGB patients with 150 cm BPL reported 0.2% revisions for malnutrition, 0.4% for GJ stenosis, and 4.7% for reflux [38]. The longest follow-up, 20 years, by Almuhanna et al., stratified BPL by BMI (150 cm for BMI 35 kg/m^2^ with 10-cm increments per BMI point) and reported a 5.1% overall revision rate, with malnutrition accounting for 2.29% [39].

Our findings indicate that complication rates following OAGB in our cohort are higher than those reported in the YOMEGA trial, suggesting similar outcomes when standardised limb lengths and follow-up protocols are applied. Significantly higher complication rates were observed when compared with the UK MGB Collaborative, a large multicentre cohort. This discrepancy likely reflects differences in sample size, patient selection, biliopancreatic limb length variability, and heterogeneity in surgical technique and postoperative surveillance.

The higher rates of severe protein malnutrition and reflux observed in comparison with Carbajo et al. may be attributable to differences in limb-length configuration and nutritional monitoring strategies. Collectively, these results emphasise the critical role of individualised limb-length selection and rigorous long-term nutritional follow-up to mitigate severe complications after OAGB.

Boyle et al. found no significant difference in outcomes between 150 and 200 cm BPL length [40]. Our findings of high complication and revision rates with a 250 cm BPL support limiting the BPL to < 250 cm, likely 150–180 cm, to reduce adverse outcomes while producing an acceptable clinical gain. We recommend measuring the common channel in all patients planned for BPL ≥ 180 cm. The recommendation to avoid BPL ≥ 250 cm, while reasonable as a hypothesis, is in need of more supporting data from future studies.One important conclusion of our study suggest that all patients who had OAGB with 250 cm of BPL or more need close long-term follow up to early diagnose and manage similar complications reported by this study.

## Strengths and Limitations

The most important strength of the study being a multi-centre project. All the data were captured for the included cohort of 97 patients. Final analysis was conducted only for 78 patients who completed a mean follow-up of 11 years (7–15 years). The study is showing serious and high-rate complications of BPL of 250 cm, a result that is likely to change the practice, prevent harm to the patients and help surgeons worldwide to tailor the OAGB procedure and never exceed 200 cm BPL. This is likely to improve quality of life, avoid major long-term complications and reduce the need for revisions and diminish the cost and financial burden on the patients and the health systems.

The following points are to be taken with caution:

1. Limited patient cohort: Although the study includes a total of 892 OAGB procedures, only 97 patients (10.9%) underwent the procedure with a BPL of 250 cm. A sample size of 97 patients may not fully represent the broader population who undergo this procedure, limiting the generalizability of the results.
2. Follow-up loss: The study had an attrition rate of 19.6%. This loss can lead to a selection bias and potentially an underreporting of complications, as patients with severe or life-threatening issues might be more likely to drop out of follow-up, miss appointments or be treated at different institutions.
3. Lack of control group: The study did not include a control group of patients who underwent OAGB with a different BPL length, although it referred to some studies with comparable BPL and follow-up.
4. Potential confounding variables: The study does not discuss the control of potential confounding variables such as dietary habits, pre-existing gastrointestinal conditions (like hiatus hernia), and patient compliance with post-surgery care. These factors could influence the outcomes and complications, but were not systematically controlled for in the analysis.
5. Bias in reporting complications: The study mainly focuses on severe complications that required revisions. However, milder complications or those that did not result in revisions might have been underreported or overlooked, leading to a potential overestimation of the success rate or revision rate. Our true complication rates may be higher due to relocation, severe complications or unregistered deaths.
6. Consistency of surgical practices: all operations were conducted by consultant bariatric surgeons. Surgical techniques, such as the length of the gastric pouch, size of the gastrojejunostomy, and orientation of the anastomosis, might differ among surgeons, leading to variability in complication rates. The study does not account for these variations.
7. No standardised protocol for revisions: Although severe complications are detailed, the criteria for when a revision was deemed necessary are not fully standardised. Different surgeons or medical teams may have different thresholds for when to revise a patient, leading to potential bias in the revision rates. This study is trying to form a baseline guidance on how to deal with liver failure, protein malnutrition, diarrhoea and reflux after OAGB.
8. Long-term outcomes and lack of randomised trials: The study lacks comparison with other surgical approaches through randomised controlled trials (RCTs), which would help establish the causal relationship between the 250 cm BPL and the complications observed. Most of the existing studies mentioned in the discussion are also retrospective, making the evidence less robust.
9. Limited generalizability to specific populations: The study included patients aged 20–65 years with a BMI of 31–73.2 kg/m^2^. The findings may not apply to younger or older populations, or those with extremely high or low BMIs, which may require different surgical approaches or have different risks associated with the procedure.
10. Potential incomplete reporting of long-term effects: Although the follow-up period ranged from 7–15 years, the study does not fully explore long-term quality-of-life outcomes, mental health effects, or other secondary conditions that might arise from the surgery. These aspects could provide additional insights into the overall impact of the procedure.
11. The absence of a control or comparator group significantly weakened the causal inference regarding limb length. Due to a multicentre study design, it was not possible to have a matched comparative group within the time frame to produce this work.
12. Absence of common channel measurement: A 250 cm BPL in a patient with an 900 cm total small bowel length (TSBL) leaves a massive 650 cm common channel. Same 250 cm BPL in a patient with a 400 cm TSBL leaves a critically short 150 cm common channel.No common channel measurement was conducted for most of the patients, and therefore it is interesting to know what common channel length exhibits any malnutrition or diarrhoea in future studies.

## Conclusions

In this multicentre retrospective cohort study with long-term follow-up, one-anastomosis gastric bypass performed with a fixed biliopancreatic limb length of 250 cm was associated with an unacceptably high incidence of severe late complications requiring revisional surgery.

The predominant complications were malabsorptive and reflux-related, including protein–calorie malnutrition, chronic diarrhoea, liver failure, and refractory gastro-oesophageal reflux. These findings highlight the importance of careful tailoring of biliopancreatic limb length in OAGB and support the avoidance of routinely long limbs without consideration of total small bowel length and common channel preservation.

While OAGB remains an effective bariatric and metabolic procedure, excessive malabsorption may expose patients to significant long-term morbidity. Prospective studies and standardised long-term follow-up protocols are required to better define optimal limb lengths and to balance durable weight loss against nutritional safety.

## Data Availability

No datasets were generated or analysed during the current study.

## Abbreviations

OAGB: One anastomosis gastric bypass
RYGB: Roux En-y Gastric bypass
LSG: Laparoscopic sleeve gastrectomy
BPL: Biliopancreatic limb Length, Revisions
